# Gene Expression Response of *Trichophyton rubrum* during Coculture on Keratinocytes Exposed to Antifungal Agents

**DOI:** 10.1155/2015/180535

**Published:** 2015-07-14

**Authors:** Tatiana Takahasi Komoto, Tamires Aparecida Bitencourt, Gabriel Silva, Rene Oliveira Beleboni, Mozart Marins, Ana Lúcia Fachin

**Affiliations:** Unidade de Biotecnologia, Universidade de Ribeirão Preto, Avenida Costábile Romano 2201, 14096-900 Ribeirão Preto, SP, Brazil

## Abstract

*Trichophyton rubrum* is the most common causative agent of dermatomycoses worldwide, causing infection in the stratum corneum, nails, and hair. Despite the high prevalence of these infections, little is known about the molecular mechanisms involved in the fungal-host interaction, particularly during antifungal treatment. The aim of this work was to evaluate the gene expression of *T. rubrum* cocultured with keratinocytes and treated with the flavonoid *trans*-chalcone and the glycoalkaloid *α*-solanine. Both substances showed a marked antifungal activity against *T. rubrum* strain CBS (MIC = 1.15 and 17.8 *µ*g/mL, resp.). Cytotoxicity assay against HaCaT cells produced IC_50_ values of 44.18 to *trans*-chalcone and 61.60 *µ*M to *α*-solanine. The interaction of keratinocytes with *T. rubrum* conidia upregulated the expression of genes involved in the glyoxylate cycle, ergosterol synthesis, and genes encoding proteases but downregulated the ABC transporter *Tru*MDR2 gene. However, both antifungals downregulated the ERG1 and ERG11, metalloprotease 4, serine proteinase, and *Tru*MDR2 genes. Furthermore, the *trans*-chalcone downregulated the genes involved in the glyoxylate pathway, isocitrate lyase, and citrate synthase. Considering the urgent need for more efficient and safer antifungals, these results contribute to a better understanding of fungal-host interactions and to the discovery of new antifungal targets.

## 1. Introduction

*Trichophyton rubrum* is the most prevalent dermatophyte, accounting for about 80% of dermatomycoses that affect keratinized tissues such as skin, hair, and nails [1]. Moreover, these infections play an important role in the scenario of opportunistic infections in immunocompromised patients [2]. Despite the high prevalence, little is known about the molecular mechanisms involved in the fungal pathogen-host interaction, much less when the infection is treated with antifungal agents.

For a better understanding of this process, culture medium supplemented with keratin and coculture of* T. rubrum* on human keratinocytes have been used to mimic skin infection with dermatophytes. The results of gene expression analysis have shown the involvement of important genes in the process of infection, which may contribute to the discovery of new antifungal targets and subsequent new antifungal agents [3–5].

The understanding of the mode of action of antifungal agents, such as the flavonoid* trans*-chalcone and the glycoalkaloid *α*-solanine, is important since these compounds can act on different specific targets of the fungus, a fact that could render these compounds more effective against resistant strains. In a screening study of natural compounds inhibiting the enzyme fatty acid synthase,* trans*-chalcone was the flavonoid exhibiting the best antifungal activity against* T. rubrum* strain MYA 3108, reducing ergosterol levels and affecting fatty acid synthesis [6]. Pinto et al., 2011, [7] demonstrated expressive antifungal activity of glycoalkaloids. In fact, solamargine and solasonine exhibited strong activity against dermatophytic fungi, including* T. rubrum*. A major mode of antifungal action of glycoalkaloids is the alteration of cell membrane integrity through binding to 3-beta-hydroxy sterol groups, causing an increase in membrane permeability, pore formation, and the consequent breakdown and loss of cell integrity [8].

The aim of this work was to evaluate the gene expression modulation of target genes in* T. rubrum* in response to coculture on human keratinocytes and coculture exposed to the antifungal agents* trans*-chalcone and *α*-solanine. To our knowledge, this is the first study describing the modulation of gene expression in* T. rubrum* during the interaction with keratinocytes exposed to antifungal compounds.

## 2. Methods

### 2.1. Strains, Media, and Growth Conditions

*Trichophyton rubrum strain CBS 118892 *was cultured on Sabouraud dextrose agar (Oxoid, Hampshire, England) for 15 days at 28°C until suitable conidiation.

### 2.2. Chemicals

*Trans*-chalcone and *α*-solanine were purchased from Sigma-Aldrich (St. Louis, MO, USA) and diluted in 10% aqueous DMSO. Terbinafine was purchased from Sigma-Aldrich (St. Louis, MO, USA) and diluted in 5% aqueous DMSO. The final concentration of DMSO used in the antifungal and coculture assays was fixed at a maximum of 0.5%. Terbinafine was used as positive control (commercial antifungal involved in ergosterol synthesis inhibition) [9]. Negative controls consisted of DMSO without the tested compounds at a final concentration of 0.5%.

### 2.3. Determination of Minimum Inhibitory Concentration and Minimum Fungicidal Concentration

Susceptibility of* T. rubrum* strain CBS 118892 (1.0 to 3 × 10^5^ CFU/mL) was assessed by determining the minimum inhibitory concentration (MIC) of different concentrations of* trans*-chalcone, *α*-solanine, and terbinafine, using the M38-A microdilution technique proposed by the Clinical and Laboratory Standards Institute [10] and described by Bitencourt et al. [6] The MIC_100_ was defined as the lowest concentration of the drug that completely inhibited the growth of* T. rubrum*. Microtiter trays were incubated at 28°C and MICs were recorded after 7 days of incubation.

The minimum fungicidal concentration (MFC) was determined from the results of the MIC tests. An aliquot of 100 *μ*L of the respective wells in which the tested concentrations of the antifungals showed inhibition was seeded in duplicate onto the surface of plates containing Sabouraud dextrose agar and incubated with* T. rubrum* for 24 h at 28°C. The MFC was defined as the lowest concentration of the tested drug that inhibited fungal growth. The MIC and MFC assays were carried out in three independent experiments performed in triplicate.

### 2.4. Keratinocytes, Media, and Growth Conditions

The cell line of immortalized human keratinocytes (HaCaT) was provided by Professor Dr. Ana Paula de Souza Pardo (University of Dentistry of Piracicaba, UNICAMP) and Professor Dr. João Ernesto de Carvalho (Division of Pharmacology and Toxicology, Multidisciplinary Center for Chemical, Biological and Agricultural Research, CPQBA). Keratinocytes were cultured in RPMI medium (Sigma) supplemented with 10% fetal bovine serum (FBS) at 37°C in a humidified atmosphere containing 5% CO_2_. Penicillin (100 U/mL) and streptomycin (100 *μ*g/mL) were added to the medium.

### 2.5. Cytotoxicity Testing of the Antifungal Agents

The keratinocytes were cultured in RPMI medium at 37°C in a humidified atmosphere containing 5% CO_2_ until 90% confluence. The cells were trypsinized (0.15% trypsin and 0.02% EDTA), counted in a hemocytometer (2.5 × 10^5^ cells/well), and incubated in a 96-well plate for 24 h. A stock solution (10 mg/mL) of the antifungal agents was prepared in 10% DMSO and each agent was directly diluted in RPMI medium. After addition of the antifungals in fresh medium, the cells were cultured at 37°C in a 5% CO_2_ atmosphere for 24 h and cytotoxicity was analyzed by the MTT assay [11], with modifications, as described by Rizo et al., 2013 [12]. Briefly, 20 *μ*L MTT/well (5 mg/mL in Hanks solution) was added to the 96-well plate and incubated for 4 h under the same conditions. All treatments were carried out in triplicate. The formazan dye was extracted with 200 *μ*L isopropanol and quantified spectrophotometrically at 550 nm (BioTek ELx800, Singapore). The absorbance of untreated cells was used as a reference.

### 2.6. Coculture Conditions and Exposure to Natural Compounds

Keratinocytes were collected, washed, and counted in a hemocytometer. A total of 2.5 × 10^5^ cells/mL were plated on RPMI plus 2% FBS in 250 mL tissue culture flasks and cultured for 24 h at 37°C in 5% CO_2_. The* T. rubrum* solution (1 × 10^7^ conidia/mL) was grown in 5 mL liquid Sabouraud medium for 7 h under gentle shaking. The keratinocyte culture was recovered by centrifugation, washed in saline, and resuspended in RPMI plus 2% FBS. The conidia solution and 0.288 *μ*M* trans*-chalcone or 4.32 *μ*M *α*-solanine or 0.0162 *μ*M terbinafine were added to the keratinocyte cultures and incubated for 24 h at 37°C in 5% CO_2_. One control and one coculture flask per treatment were reserved for staining with May-Grünwald and Giemsa for verification of infection. Fungi and human cells were recovered by scraping and centrifuged at 1,730 g for 10 min. RNA was prepared directly from the recovered cells as described in the next item.

### 2.7. RNA Isolation

The coculture was treated with lysis solution (20 mg/mL of lysing enzymes from* Trichoderma harzianum* purchased from Sigma-Aldrich; 0.7 M KCl and 1 M MgSO_4_, pH 6.8) for 1 h at 28°C under gentle shaking, followed by centrifugation at 1,000 g for 10 min. Total RNA was extracted from the coculture using the Illustra RNAspin Mini RNA Isolation Kit (GE Healthcare). The RNA preparations were analyzed for the absence of proteins and phenol by UV spectrophotometry, while RNA integrity was confirmed by denaturing electrophoresis. The RNA samples were used for RT-PCR. Uninfected cultured keratinocytes and* T. rubrum* conidia were included as controls.

### 2.8. Quantitative RT-PCR

A set of nine genes were selected to evaluate the level of gene expression in* T. rubrum* cocultivated with keratinocytes in presence or absence of natural compounds by quantitative RT-PCR (Table 1). The genes were chosen based on their potential as antifungal targets such as glyoxylate pathway [13], ergosterol biosynthesis [14, 15], response to drugs [16], and proteases related to the infection process [5]. Complementary DNA was synthesized from 500 ng total RNA in a 20 *μ*L reaction volume using the RevertAid H Minus First Strand cDNA Synthesis Kit (Fermentas). Quantitative RT-PCR experiments were performed in duplicate using the SYBR Taq ReadyMix Kit (Sigma) in an Mx3300 QPCR system (Stratagene). The cycling conditions were initial denaturation at 94°C for 10 min, followed by 40 cycles at 94°C for 2 min, at 60°C for 60 s, and at 72°C for 1 min. A dissociation curve was constructed at the end of each PCR cycle to verify single product amplification. Gene expression levels were calculated by the comparative Ct method using beta-tubulin as normalizer gene [17] and* T. rubrum* conidia and* T. rubrum* coculture without the tested drugs as reference. The results are reported as the mean ± standard deviation of two experiments.

### 2.9. Data Analysis

The cytotoxicity results were analyzed by two-way ANOVA (*p* < 0.05). IC_50_ values were calculated by nonlinear regression analysis.

## 3. Results 

### 3.1. Antifungal and Cytotoxic Activity of the Inhibitory Compounds

*Trans*-chalcone (MIC = 1.15 *μ*M) and *α*-solanine (MIC = 17.28 *μ*M) exhibited marked inhibitory activity against* T. rubrum*. The compounds *α*-solanine and terbinafine exerted fungicidal activity against the CBS 118892 strain, while* trans*-chalcone exhibited fungistatic activity (Table 2). Cytotoxicity was evaluated by culturing keratinocytes for 24 h in the presence of the tested drugs and the IC_50_ results are shown in Table 2.* Trans*-chalcone and *α*-solanine exhibited greater cytotoxicity against the mammalian cells than the commercial drug terbinafine but did not compromise interpretation of the results of the coculture assays of* T. rubrum* on keratinocytes.

### 3.2. Coculture of* T. rubrum* Exposed to the Natural Compounds

**Figure 1:**
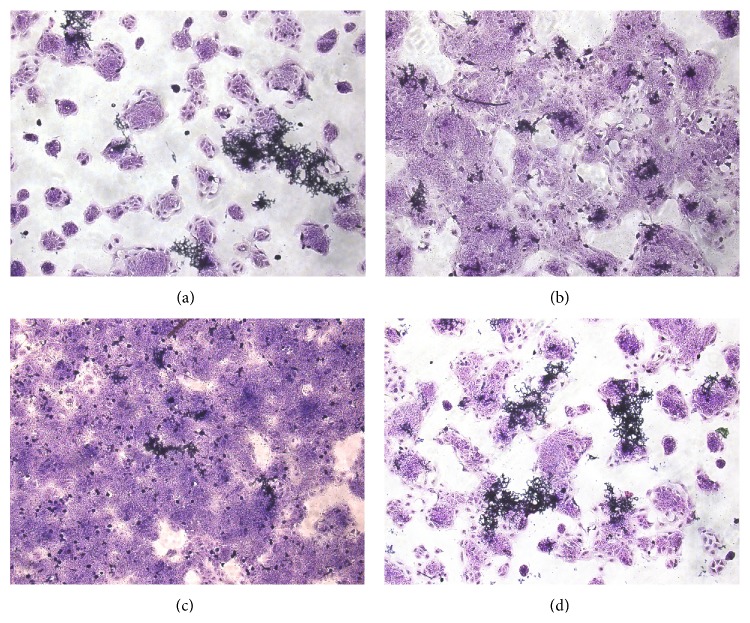
Coculture of* Trichophyton rubrum* in HaCaT cells exposed to antifungal agents. The* T. rubrum* solution (1 × 10^7^ conidia/mL) was preincubated in Sabouraud medium for 7 h, 2.5 × 10^5^ keratinocytes/mL were added, and the culture was exposed to the antifungal agents for 24 h. (a) Control; (b) 0.0162 *μ*M terbinafine; (c) 4.32 *μ*M *α*-solanine; (d) 0.288 *μ*M* trans*-chalcone.

Figure 1 illustrates the results obtained for coculture of* T. rubrum* on keratinocytes and in the presence of the drugs tested. The hyphae were found to be fragmented in the presence of *α*-solanine. The same was* observed* for terbinafine, while hyphae were twisted in the presence of* trans*-chalcone.

### 3.3. Modulation of Gene Expression

#### 3.3.1. Response of* T. rubrum* to Keratinocytes

**Figure 2:**
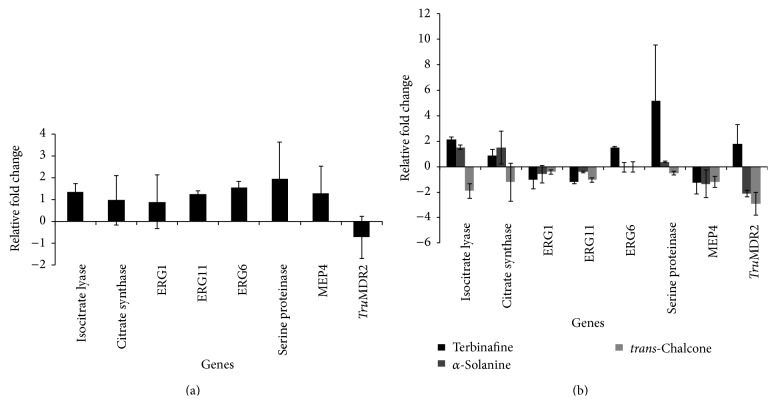
(a) Modulation of gene expression in* Trichophyton rubrum* cocultured with HaCaT cells for 24 h. The reference used was the conidia solution. (b) Modulation of gene expression in* T. rubrum* cocultured with HaCaT cells exposed to 0.0162 *μ*M terbinafine, 4.32 *μ*M *α*-solanine, and 0.288 *μ*M* trans*-chalcone for 24 h. The reference used was the coculture without antifungals.

Figure 2(a) shows the effect of keratinocytes on gene expression modulation in* T. rubrum*. Significant induction of the genes involved in the glyoxylate cycle and ergosterol synthesis and of the genes encoding proteases was observed. In contrast, the* Tru*MDR2 gene, involved in multiple drug resistance, was repressed.

#### 3.3.2. Response of* T. rubrum* to Keratinocytes Exposed to the Antifungal Agents

The results of gene expression analysis of the* T. rubrum* coculture exposed to the antifungal agents are shown in Figure 2(b). The genes involved in the glyoxylate pathway, isocitrate lyase and citrate synthase, were induced in the presence of *α*-solanine and terbinafine. In contrast,* trans*-chalcone repressed these target genes. With respect to the genes involved in ergosterol synthesis,* trans*-chalcone, *α*-solanine, and terbinafine inhibited the expression of the ERG1 and ERG11 genes. The ERG6 gene was induced by terbinafine, while neither* trans*-chalcone nor *α*-solanine modulated the expression of this gene.

Regarding the modulation of the genes encoding proteases, the flavonoid* trans*-chalcone repressed the expression of serine proteinase (subtilisin). Furthermore, a strong induction of this gene was observed in the presence of terbinafine and, to a lesser extent, in the presence of *α*-solanine. On the other hand, repression of the MEP4 gene was observed when the fungus was cultured in the presence of terbinafine, *α*-solanine, and* trans*-chalcone. Terbinafine induced the expression of the* Tru*MDR2 gene. In contrast,* trans*-chalcone and *α*-solanine repressed the expression of this gene (Figure 2(b)).

## 4. Discussion 

Chalcones are an important group of natural compounds that possess a broad range of biological activities, including antifungal activity [18–22]. In this respect, Boeck et al. [23] showed the chalcones tested in their study were effective against the series of dermatophytes* Microsporum canis*,* Epidermophyton floccosum*, and* Microsporum gypseum*. The chalcone 3-(2-chlorophenyl)-1-(2-hydroxy-4,6-dimethoxyphenyl)prop-2-en-1-one inhibited all clinical strains of* T. rubrum* isolated from skin infections of immunocompromised patients, with MIC_90_ ranging from 12.5 to 25 *μ*g/mL. In contrast, the authors observed that none of the chalcones tested was effective against the yeast species* Candida albicans*,* Saccharomyces cerevisiae*, or* Cryptococcus neoformans*, nor against the filamentous fungi* Aspergillus niger*,* A. fumigates*, or* A. flavus*. Furthermore, results from our research group showed that* trans*-chalcone exerted no antifungal activity against aflatoxigenic* Aspergillus flavus* strains (MIC_100_ > 1,000 *μ*g/mL) (unpublished data). Also in the present study,* trans*-chalcone exhibited marked activity against* T. rubrum* (MIC = 1.15 *μ*M), suggesting that this flavonoid exhibits specific activity against dermatophytes, which is supported by previous data.

*α*-Solanine was also effective against* T. rubrum* (MIC = 17.28 *μ*g/mL). Much of the biological activity of glycoalkaloids isolated from different* Solanum* species is due to their ability to disrupt sterol-containing membranes in erythrocytes and fungal protoplasts [24]. Other mechanisms of action have been reported for two important glycoalkaloids found in potatoes (*α*-solanine and chaconine), which exert antifungal activity by inhibiting sporulation and hyphal growth, in addition to causing hyphal distortion. Furthermore, data suggest that these glycoalkaloids can inhibit the biosynthesis of essential compounds or even act as antimetabolites [25].

In the present study, the isocitrate lyase and citrate synthase genes involved in the glyoxylate pathway were induced when* T. rubrum* was cocultured on keratinocytes, suggesting that this pathway is important for the process of infection caused by dermatophytes. In addition to the absence of the glyoxylate cycle in mammals, metabolic pathways are currently being explored as potential antifungal targets since they contribute to metabolic flexibility by permitting microbial cells to survive in nutrient-limited host niches during infection [26]. The glyoxylate cycle permits some microorganisms to synthesize glucose from lipids and other alternative carbon sources. Since dermatophytes specifically infect epidermal structures rich in keratin and lipids, the functional characterization of genes involved in the glyoxylate cycle should contribute to the understanding of the virulence of these fungi in the host and also to the discovery of interesting pharmacological targets [26]. The importance of three enzymes of the glyoxylate cycle, isocitrate lyase, malate synthase, and citrate synthase for fungal virulence has been demonstrated in* Candida albicans* [27]. In* C. albicans*, metabolic flexibility is important for adaptation and survival of the fungus in host niches and also for biological parameters of pathogenicity and virulence [13, 28–30].

The ERG1 and ERG11 genes encode, respectively, the squalene epoxidase and lanosterol C-14 *α*-demethylase, the main target of terbinafine and currently used azolic antifungal agents [9, 31]. In the present study, *α*-solanine and* trans*-chalcone inhibited the expression of the ERG1 and ERG11 genes. In fact,* trans*-chalcone seems to interfere with ergosterol synthesis in the cell, as indicated by previous studies showing a reduction of 74% and 77% in ergosterol levels when a wild-type and a mutant (Δ*Tru*MDR2) strain of* T. rubrum* were cultured in the presence of 0.5 MIC of* trans*-chalcone. At the MIC of* trans*-chalcone, ergosterol content was reduced by 100% in the two strains [6].

Neither* trans*-chalcone nor *α*-solanine repressed the expression of the ERG6 gene. This finding might be due to repression of the ERG11 gene which encodes the main enzyme responsible for the first step of ergosterol biosynthesis, impairing activation of the remaining pathway. The ERG6 gene (sterol methyltransferase) encodes an enzyme that participates in the ergosterol pathway, converting zymosterol to fecosterol. However, data suggest that ERG6 is not the main target gene of the pathway, since no differences in vegetative growth were observed in an ERG6 null mutant strain of* Saccharomyces cerevisiae* and neither meiosis nor sporulation was affected, demonstrating that this gene is not essential for this fungus. However, this gene can affect different phenotypic characteristics such as membrane permeability and fluidity, sensitivity to cycloheximide, the capacity of genetic transformation, and tryptophan uptake [32].

Infection with dermatophytes requires installation of the pathogen and the adherence of arthroconidia to the epithelial surface, followed by their germination and penetration of hyphae into the stratum corneum of skin. After adhesion, dermatophytes require nutrients for their development and survival. However, it is known that membrane permeability prevents carbon, nitrogen, phosphorus, and sulfur compounds from being transported into cells, and enzymes able to degrade and transform these macromolecules into smaller molecules are therefore necessary. Different enzymes such as proteases, lipases, elastases, and collagenases are produced during this process [33, 34].

The main families of proteases secreted by fungi are subtilisins (serine proteases) and fungalisins (metalloproteases) [35]. Studies on* Microsporum canis* and* T. rubrum* have shown that metalloproteases and serine proteinases are potential virulence-related factors in dermatophytes [36, 37]. Zhang et al. [38] obtained five metalloprotease mutants by* Agrobacterium tumefaciens*-mediated transformation. The authors observed a significant reduction in virulence in the MEP4 and MEP5 null mutant strains. The MEP3 mutant strain showed virulence similar to that of the wild-type strain.

Another important protease that was found to be modulated in the present study was serine proteinase, which belongs to the subtilisin family and, like metalloproteases, is classified as an endoprotease [39, 40]. Serine proteinases were the proteases most frequently isolated from culture supernatants during the growth of* T. rubrum* [41]. This protease was also detected in another experiment conducted by our group, mimicking skin in minimal culture medium containing keratin and elastin (data not shown). Induction of this protease was observed compared to the control, as well as in the present study in which the result was validated in a keratinocyte model of infection.

Involvement of the* Tru*MDR2 transporter in the transport of terbinafine, as well as of ethidium bromide compounds and 4NQO, has been demonstrated by Fachin et al. [16] in assays testing the susceptibility of the Δ*Tru*MDR2 mutant to these drugs. Indeed, Zhang et al. [42] observed in the microarray experiment that terbinafine induced the transcription of multidrug-resistance (MDR) genes, including DW686642,* Tru*MDR2, and MDR1 of* T. rubrum*. In the present study, despite the fact that terbinafine showed antifungal activity and low cytotoxicity against the cells of keratinocytes, terbinafine induced the expression of the* Tru*MDR2 gene. In contrast, the expression of the* TruMDR2* gene was repressed in the presence of* trans*-chalcone and *α*-solanine. This finding makes these compounds even more interesting since inhibition of the efflux of these drugs by the cell may impair the development of resistance to these inhibitors in* T. rubrum*. Furthermore, Bitencourt et al. [6] observed that the Δ*Tru*MDR2 mutant strain is more sensitive to* trans*-chalcone (MIC = 1.9 *μ*g/mL) than another* T. rubrum* strain (MYA3108) (MIC = 7.5 *μ*g/mL). Considering the involvement of the* TruMDR2* gene both in the mechanism of drug transport [16] and in the pathogenicity of* T. rubrum* [41], this gene is an important target to screen for novel antifungal agents.

## 5. Conclusion

*Trans*-chalcone exhibited significant antifungal activity and was moderately cytotoxic to keratinocytes. The interaction between* T. rubrum* and keratinocytes induced the expression of genes involved in glyoxylate cycle and ergosterol synthesis, as well as genes encoding proteases. However, gene expression analysis demonstrated that* trans*-chalcone repressed the expression of virulence-related genes (isocitrate lyase, citrate synthase, and MEP4), target genes of commercial antifungal (ERG1 and ERG11), and also the* Tru*MDR2 transporter involved in multiple drug resistance. These findings render* trans*-chalcone an interesting compound in future studies, especially its application to resistant strains.

## Figures and Tables

**Table 1:** Primers used for RT- PCR.

Gene	Sequence	Size (bp)	Reference
Isocitrate lyase	F: 5′-ACAACCTCTCGCCTTCATTC-3′ R: 5′-GGTCAGATATCAGGGCAGTTG-3′	144	This paper
Citrate synthase	F: 5′-GAGGAGGGTATTCGCTTCCG-3′ R: 5′-CGAACTTGTTGCTCGGTTGG-3′	143	This paper
Metalloprotease MEP4	F: 5′-GCATGGACTTATGCTTGCGG-3′ R: 5′-TGGATATCTGGGGAAGGCGA-3′	131	This paper
Serine proteinase	F: 5′-GCTGGCTCCAATCTACTCATAC-3′ R: 5′-CGCTGTATCCCTTCATCTTGT-3′	105	This paper
ERG1	F: 5′-GTGAAGATACCTTTCCCTAGCG-3′ R: 5′-TTATGGTAGAAACGGCCTTGG-3′	148	This paper
ERG6	F: 5′-CTCTGGCAAGACACGAACAC-3′ R: 5′-CCTTGCAGCCGGTGAAGG-3′	126	[6]
ERG 11	F: 5′-CACTTCCTTGCCCTGTAGAGATC-3′ R: 5′-GGAGTTTTCAATGTCAGCAAGGTTT-3′	78	[15]
*Tru*MDR2	F: 5′-GCACTGATCTGCAGCTCGACC-3′ R: 5′-CCAACGTCATCCTCCCAGAC-3′	91	[16]
Beta-tubulin	F: 5′-AACATGATGGCTGCCACTGA-3′ R: 5′-AAGATGGCAGAGCAGGTAAGGT-3′	253	[17]

**Table 2:** Minimum inhibitory concentration (MIC) and minimum fungicidal concentration (MFC) of the antifungal agents against *Trichophyton rubrum* strain CBS 118892 (*µ*M). IC_50_ (*µ*M) of the antifungal agents in a human keratinocyte cell line (HaCaT).

Compound	MIC	MFC	IC_50_
*Trans*-chalcone	1.15	2.30	44.18
*α*-Solanine	17.28	17.28	61.60
Terbinafine	0.065	0.065	315.7
